# Molecular characterization of Chikungunya virus isolates from clinical samples and adult *Aedes albopictus *mosquitoes emerged from larvae from Kerala, South India

**DOI:** 10.1186/1743-422X-7-189

**Published:** 2010-08-13

**Authors:** Kudukkil P Niyas, Rachy Abraham, Ramakrishnan Nair Unnikrishnan, Thomas Mathew, Sajith Nair, Anoop Manakkadan, Aneesh Issac, Easwaran Sreekumar

**Affiliations:** 1 Molecular Virology Laboratory, Rajiv Gandhi Centre for Biotechnology (RGCB), Thycaud P.O., Thiruvananthapuram-695014, Kerala, India; 2 State Disease Control and Monitoring Cell (SDCMC), National Rural Health Mission (NRHM), Government of Kerala, Thiruvananthapuram-695014, Kerala, India; 3 Department of Community Medicine, Medical College, Thiruvananthapuram, Kerala, India

## Abstract

Chikungunya virus (CHIKV), an arthritogenic alphavirus, is transmitted to humans by infected *Aedes (Ae.) aegypti *and *Ae.albopictus *mosquitoes. In the study, reverse-transcription PCR (RT PCR) and virus isolation detected CHIKV in patient samples and also in adult *Ae.albopictus *mosquitoes that was derived from larvae collected during a chikungunya (CHIK) outbreak in Kerala in 2009. The CHIKV strains involved in the outbreak were the East, Central and South African (ECSA) genotype that had the E1 A226V mutation. The viral strains from the mosquitoes and CHIK patients from the same area showed a close relationship based on phylogenetic analysis. Genetic characterization by partial sequencing of non-structural protein 2 (nsP2; 378 bp), envelope E1 (505 bp) and E2 (428 bp) identified one critical mutation in the E2 protein coding region of these CHIKV strains. This novel, non-conservative mutation, L210Q, consistently present in both human and mosquito-derived samples studied, was within the region of the E2 protein (amino acids E2 200-220) that determines mosquito cell infectivity in many alpha viruses. Our results show the involvement of *Ae. albopictus *in this outbreak in Kerala and appearance of CHIKV with novel genetic changes. Detection of virus in adult mosquitoes, emerged in the laboratory from larvae, also points to the possibility of transovarial transmission (TOT) of mutant CHIKV strains in mosquitoes.

## Findings

Chikungunya virus (CHIKV) is an alphavirus of the *Togaviridae *family and is an important re-emerging pathogen. It has been responsible for major fever epidemics in many parts of the world [1,2]. The disease, chikungunya (CHIK), is characterized by high fever, headache, myalgia, severe and prolonged arthralgia, and erythematous skin rashes [1]. In general, it is considered as a self-limiting illness. However, recent outbreaks of CHIK exhibited unusual severity, neurological complications and suspected mortality [3-6]. The disease is transmitted by the bite of *Aedes ( Ae.) aegypti *and *Ae. albopictus *mosquitoes. Studies have shown that *Ae. albopictus *facilitates rapid transmission of the new strains of CHIKV that had adaptive mutations in the viral genome [7,8].

CHIK epidemic has caused considerable morbidity in recent years in India [9,10]. Kerala, in South India, was one among the worst affected states [11-14]. Abundance of *Ae.albopictus *in many parts of the state was implicated for the rapid spread of the infection [11]. Recent studies carried out in CHIKV from Kerala [11,12,14] have revealed novel genetic changes in the virus isolates from 2006-2008 outbreaks. Reports on virus isolation from mosquito vectors from the region are currently not available. The aim of the present work was to look for novel genetic changes in the isolates from 2009 by sequence analysis of selected genomic regions, and also to look for CHIKV in *Ae. albopictus *mosquitoes

**Figure 1:**
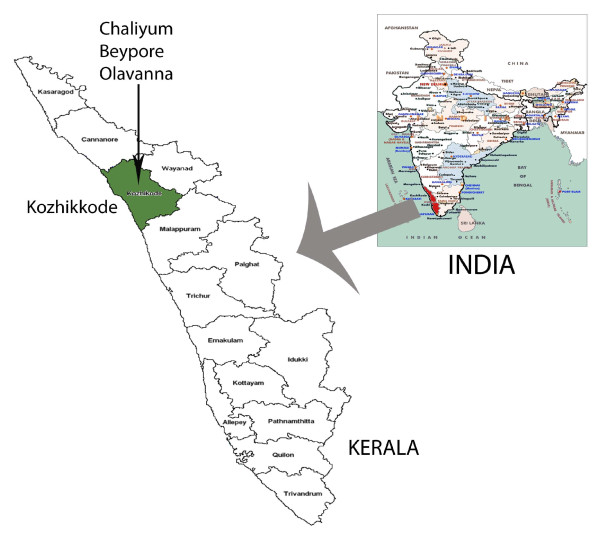
**Map of Kerala showing the location of sample collection areas**.

The study was done during a fever outbreak in May-September 2009 in Kozhikkode district of northern Kerala (Figure 1). All the patients included in the study had classical symptoms of CHIK [15]. Samples were obtained from the outpatient department of three Primary Health Centres (Olavanna, Beypore and Chaliyum) in the district. 2-5ml of whole blood was collected from patients who were clinically diagnosed with CHIK and had a history of fever of 1-5 days duration. Samples were transported to the laboratory in wet-ice; serum was separated and stored in aliquots at -80°C. Standard ethical and bio-safety guidelines were followed, and informed consent was obtained from all the patients prior to blood withdrawal.

For virus detection in mosquitoes, households of CHIK patients, whose serum samples were confirmed in the laboratory by RT-PCR, were subsequently visited and larval sampling was done. Stagnant water collected in discarded articles such as coconut shells, broken earthern-wares, plastic bottles and damaged drains were searched for *Ae. albopictus *larvae. Third and fourth instar larvae and pupae were phenotypically identified *in situ *using standard keys and these were collected and transferred to containers with fresh water. Four households each in Olavanna and Chaliyum, and three households in Beypore were surveyed. Larvae and pupae collected from each location were made into a single pool. In the laboratory, these three pools were independently reared in bowls with water, kept in mosquito cages at an ambient temperature of 25-30°C and a relative humidity 60-70%. The newly emerged adult mosquitoes were collected and frozen at -20°C for 30 minutes. Whole-mosquito tissue extracts were prepared by homogenizing pools of adult mosquitoes [each pool with 30 individual mosquitoes (both males and females) representing a single location]. Frozen mosquitoes were homogenized in 700 μl of Dulbecos Modified Eagle's Medium (DMEM) using a micropestle. These were then clarified by centrifugation at 800 × *g *at 4°C and sterilized by filtering through 0.2 μM membrane filter (Millex GV, Millipore) and used for RNA isolation.

**Figure 2:**
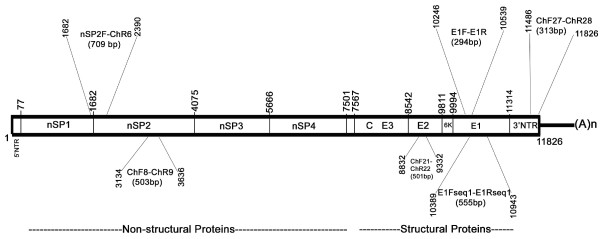
**Location of primers in the CHIKV genome**. Positions are numbered with respect to S27 sequence (GenBank Accession AF369024).

RNA isolation from the 70 patient serum samples and the three extracts from mosquito samples were carried out using QIAamp Viral RNA Mini kit (Qiagen, GmBH, Hilden) exactly as per the kit protocol. Single-step RT PCR was done using 10 μl of the isolated RNA from all the samples using Fidelitaq RT-PCR kit (USB, Cleveland, Ohio), as previously described [14]. PCR primers (Table 1; Figure 2) for CHIKV detection PCR were designed based on earlier reports [16] and on the conserved genomic regions of local strains of CHIKV [14]. The conditions for RT PCR were: a reverse transcription step at 50°C for 45 min; followed by 35 cycles of thermal cycling, which included denaturation at 95°C for 1 min, annealing at 55°C for 1 min, and an extension at 68°C for 2 min. Extreme care was taken to avoid PCR-contamination, by carrying out the pre-and post amplification steps in laboratories located in separate buildings and also by including a non-template control in all amplifications.

**Table 1:** Details of the primers used for PCR amplification in the study.

Primer Name	**Sequence (5'→3'); location with respect to S27 sequence (GenBank Accession **AF369024**)**	Target	**T**_**a**_ (°C)	Amplicon size	Reference
**RT PCR for CHIKV detection in patient and adult mosquitoes derived from larvae**					

E1 F	tacccatttatgtggggc (10246-10263)		52	294bp	[16]
E1 R	gcctttgtacaccacgatt (10539-10521)	E1			
NSP2F	tgccatgggaataatagagactccg (1682-1699)				
ChR6	gcgagtcaaccgtacgtgcag (2390-2370)	nsP2	55	709bp	This study
ChF27	gtcccctaagagacacattg (11486-11505)				
ChR28	tacgtccctgtgggttcggagaat (11798-11780)	3'NTR	52	313bp	[14]

**RT PCR of partial sequences CHIKV genes for sequencing and phylogenetic analysis**					

E1Fseq1	gctccgcgtcctttacc (10389-10405)				
E1Rseq1	atggcgacgcccccaaagtc (10943-10924)	E1	55	555bp	This study
ChF21	gggacacttcatcctggc (8832-8849)				[14]
ChR22	acatttgccagcggaaac (9332-9315)	E2	55	501bp	
ChF8	cctatcctcgaaacagcg (3134-3151)				[14]
ChR9	gtgactctcttagtaggc (3636-3619)	nsP2	45	503bp	

**Figure 3:**
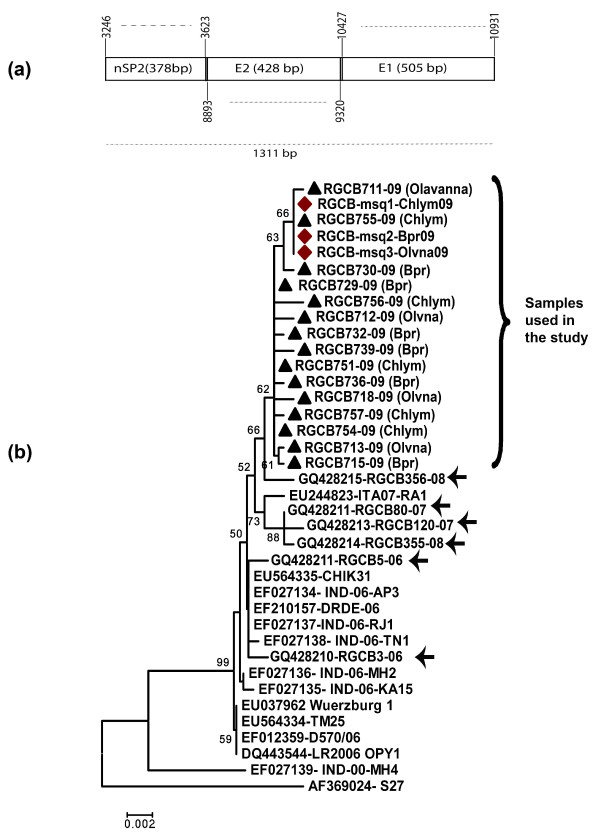
**Phylogenetic analysis of the CHIKV partial nsP2, E2 and E1 coding region nucleotide sequences**. **a) **Tandem arrangement of the sequences used for the analysis. Numbers indicate the position with respect to the sequence of S27 strain (AF369024). **b) **Neighbor-Joining Tree of corresponding sequences of CHIKV strains derived from human clinical samples constructed with 10,000 bootstrap replications. The human and mosquito sequences obtained from the study are marked 'black triangle' and 'black diamond', respectively. GenBank accession numbers and strain names are indicated. Scale bar represents the number of substitutions/site. Sequences of recent Kerala isolates are indicated by '→'.

For nucleotide sequencing and phylogenetic analysis, 15 clinical samples and all the three mosquito-derived samples were used. Only clinical samples that gave a high intensity amplicon in the primary detection PCR were selected to ensure that sufficient DNA would be available for sequencing reactions. Five clinical samples each from Olavanna, Beypore and Chaliyum were used, making a total of 15 samples. Selected regions of the CHIKV genome (nucleotide position, with respect to S27 reference sequence AF369024: nsP2 3134-3636; E2 8832-9332; E1 10246-10539; Table 1; Figure 2) were amplified by RT PCR as described above using new sets of primers (Table1; Figure 2). These specific regions were chosen as they showed nucleotide variability and novel mutations in our previous study with the local strains of CHIKV [14], making them suitable for phylogenetic analysis. Purified PCR products were directly subjected to automated DNA sequencing as per manufacturer's directions in an ABI-Prism 3730 Genetic analyzer (PE Applied Biosystems, Foster City, CA). The sequences were aligned with corresponding CHIKV sequences obtained from NCBI GenBank using Clustal W program of MEGA3.1 [17] software, with Kimura-2 distance correction. To get representation from different gene segments in the evolution of the CHIKV strains, the partial sequences of nsP2, E2 and E1 genes were arranged in tandem to obtain a 1311 bp sequence (Figure 3a), which was then used for phylogenetic analysis. The phylogeny was reconstructed by Neighbor-Joining method with 10,000 bootstrap replications using the MEGA 3.1 program. 100 μL of the mosquito extracts or patient serum samples were used for CHIKV isolation in confluent monolayer of Vero cells cultured in 75cm^2 ^flasks, as per standardized protocols [14]. The titration of CHIKV in the infected cultures was done by plaque assay using a carboxymethyl-cellulose overlay method [18] on Vero cells.

**Figure 4:**
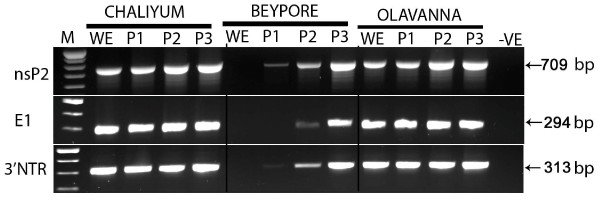
**RT PCR based detection of CHIKV RNA in adult mosquitoes derived from larvae**. WE-mosquito whole extract; P1, P2, P3-RNA from viral passage 1, 2 & 3 in Vero cells; M-molecular weight marker.

CHIKV RNA was detected in 49 out of the 70 patient samples (70%) and in adult mosquitoes derived from larvae from Chaliyum and Olavanna by RT PCR (Figure 4). All the three mosquito derived samples were positive for CHIKV, as indicated by cytopathic effects and RT PCR (Figure 4), in the 3^rd ^passage of virus isolation in Vero cell monolayer cultures. In plaque assays, the culture supernatants from these infected cells had a virus titre of 2.0 × 10^11^, 3.3 × 10^10^, 1.4 × 10^10 ^plaque forming units (pfu) ml^-1 ^for samples from Olavanna, Chaliyum and Beypore, respectively.

Analysis of the partial nucleotide sequences of nsP2 (378 bp; position 3246-3623), E1 (position 10427-10931) and E2 (position 8893- 9320) revealed a few random nucleotide changes in the CHKV isolates studied (Additional File 1) with respect to the corresponding sequences of the previous isolates from Kerala [14]. The nucleotide change T3297C observed in the 2007 & 2008 Kerala isolates, causing an L539 S mutation in the nsP2 protein, was absent in CHIKV strains of the present outbreak. A novel substitution (T3296C) was consistently observed in a few strains from patients (RGCB711, RGCB730, and RGCB755) and in all the three isolates from mosquito samples. However, this was a synonymous substitution. The E1 sequence of all the strains had the C10670T substitution resulting in the A226V mutation identified in the recent isolates of CHIKV [3,14]. Another new substitution (E1 G10864A) detected consistently in all the mosquito-derived strains and two of the clinical isolates (RGCB711 & RGCB755) can result in an amino acid change of V291I. Two nucleotide substitutions (A9114G and T9170A) were observed in the E2 coding region of all the strains studied from the outbreak. The latter substitution resulted in an amino acid change L210Q in the predicted sequence of amino acids of the E2 protein. Phylogenetic analysis revealed that the strains involved in the outbreak were closely related to the East-Central South African genotype of the CHIKV (Figure 3b). The gene sequences of CHIKV obtained from mosquito and patient samples formed a close cluster, distinct from the strains isolated previously from Kerala [14], rest of India and other parts of the world. This show a common genetic origin of the virus strains from patients and mosquitoes in this outbreak.

**Figure 5:**
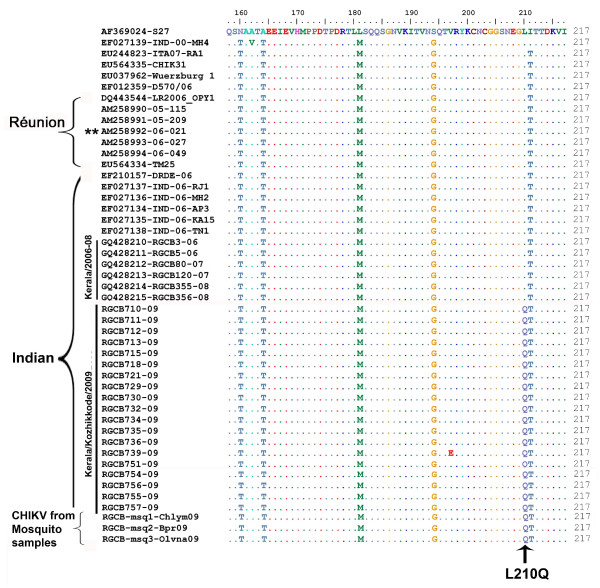
**Alignment of predicted amino acid sequences of the partial E2 protein of CHIKV strains**. The newly identified L210Q mutation in Kerala strains is indicated. The CHIKV strain from Réunion island, which was previously used in vertical transmission studies [25], is marked as '** '.

Apart from these genetic changes, an interesting observation in the study was the detection of CHIKV from adult mosquitoes derived from larval samples. Considering that these mosquitoes were freshly emerged in the laboratory from the larvae collected from areas encountering a CHIK outbreak and did not have a blood-meal, the possibility of acquiring the virus through transovarial transmission (TOT) can be thought of. Even though TOT has been proven in flaviviruses [19-23], the occurrence of this phenomenon in alphaviruses is still inconclusive [24-27]. Studies using a Réunion Island isolate of the CHIKV from 2006 outbreak [Strain 06.21; GenBank: AM258992] could not demonstrate vertical transmission in the mosquito vector [25]. The mosquito infectivity of alphaviruses is modulated by mutations in specific viral proteins [28-31]. Amino acid residues 200-220 of the E2 protein determine the cellular receptor tropism and mid-gut infectivity in *Ae. aegypti *mosquitoes [28,30]. An E2 I211T mutation was found to strongly enhance *Ae.albopictus *infectivity of CHIKV strains with the E1 A226V change [31]. Both the mutations were present in the isolates in this study and also in the recent Indian isolates [10,12,14] (Figure 5). Interestingly, the novel mutation in E2 (L210Q) that was detected exclusively in these 2009 CHIKV strains was adjacent to the E2-211 position. This substitution of the aliphatic amino acid leucine with glutamine, an amino acid with polar side chains, can have critical effects on local protein structure. One of the predicted effects of such amino acid changes is the exposure of buried protein surfaces. Possibly, this may alter the interaction of E2 with other proteins, particularly with cellular receptors, and may change the tissue tropism. However, more studies are required to understand the effects of the L210Q mutation.

The results from this study, along with the previous observations [11,12,14], indicate a constant genomic evolution of the CHIKV strains circulating in Kerala. The availability of large numbers of *Ae.albopictus *vector mosquitoes [11] and an immunologically naïve human population unexposed to CHIK in different parts of the state might facilitate recurrent infections and viral evolution. Emergence of newer strains with altered virulence and transmission potential is a possible out come of the long term viral persistence in the community. Further entomological and virological studies with these new CHIKV strains would help to understand the changing epidemiology of this re-emerging virus.

## Competing interests

The authors declare that they have no competing interests.

## Authors' contributions

KPN, SN, AM, and AI obtained patient samples, carried out RT PCR and sequencing studies. RA did the virus isolation. TM made the administrative arrangements for obtaining samples from the hospitals, and was involved in identifying CHIK patients and collecting blood samples. RNU did the collection, identification and rearing of mosquito larvae. ES conceived the study and drafted the manuscript. All authors read and approved the final manuscript.

## Supplementary Material

Additional file 1 **Clustal W alignment of the partial nucleotide sequences of Chikungunya virus nsP2, E2 and E1 protein coding region**. Click here for file
