# Supplementary material for: Molecular characterization of Chikungunya virus isolates from clinical samples and adult Aedes albopictus mosquitoes emerged from larvae from Kerala, South India
Source: Virol J. 2010 Aug 13;7:189. doi: 10.1186/1743-422X-7-189 (PMC2928196; doi:10.1186/1743-422X-7-189)
Supplement: Additional file 1 — Clustal W alignment of the partial nucleotide sequences of Chikungunya virus nsP2, E2 and E1 protein coding region. [file 1743-422X-7-189-S1.PDF]

Additional File.1 Clustal W alignment of the partial nucleotide sequences of Chikungunya virus nsP2, E2 and E1 protein coding region -positions numbered with respect to the S27 reference strain (GenBank Accession No.AF369024)

1a. nsP2 protein coding region

```
                                 3250      3260      3270      3280      3290      3300      3310      3320      3330
                              ....|....|....|....|....|....|....|....|....|....|....|....|....|....|....|....|....|....|
AF369024- S27                 GCACGCGCATGTATGGGGTGGATCTAGACAGCGGGCTATTTTCTAAACCGTTGGTGTCTGTGTATTACGCGGATAACCACTGGGATAATA
EF027139- IND-00-MH4          ..........................................................................................
EU564335-CHIK31               .T........................................................................................
EF210157-DRDE-06              .T........................................................................................
EU244823-ITA07-RA1            .T.............................................................................T..........
EU037962 Wuerzburg 1          .T........................................................................................
EF012359-D570/06              .T........................................................................................
DQ443544-LR2006_OPY1          .T........................................................................................
EU564334-TM25                 .T........................................................................................
EF027137-IND-06-RJ1           .T........................................................................................
EF027136- IND-06-MH2          .T........................................................................................
EF027134- IND-06-AP3          .T........................................................................................
EF027135- IND-06-KA15         .T........................................................................................
EF027138- IND-06-TN1          .T........................................................................................
GQ428210 RGCB3-06             .T........................................................................................
GQ428211 RGCB5-06             .T........................................................................................
GQ428212 RGCB80-07            .T.................................................C......................................
GQ428213 RGCB120-07           .T.................................................C......................................
GQ428214 RGCB355-08           .T.................................................C......................................
GQ428215 RGCB356-08           .T...................................G....................................................
RGCB710-09                    .T........................................................................................
RGCB711-09                    .T................................................C.......................................
RGCB712-09                    .T........................................................................................
RGCB713-09                    .G........................................................................................
RGCB715-09                    .A........................................................................................
RGCB718-09                    .T........................................................................................
RGCB721-09                    .T........................................................................................
RGCB729-09                    .T........................................................................................
RGCB730-09                    .T................................................C.......................................
RGCB732-09                    .T........................................................................................
RGCB734-09                    .T........................................................................................
RGCB735-09                    .T........................................................................................
RGCB736-09                    .T........................................................................................
RGCB739-09                    .T........................................................................................
RGCB751-09                    .T........................................................................................
RGCB754-09                    .T........................................................................................
RGCB755-09                    .T................................................C.......................................
RGCB756-09                    .T........................................................................................
RGCB757-09                    .T........................................................................................
RGCB-msq1-Chlym09             .T................................................C.......................................
RGCB-msq2-Bpr09               .T................................................C.......................................
RGCB-msq3-Olvna09             .T................................................C.......................................
```

```
                                3340      3350      3360      3370      3380      3390      3400      3410      3420
                            ....|....|....|....|....|....|....|....|....|....|....|....|....|....|....|....|....|....|
AF369024- S27               GGCCTGGAGGGAAGATGTTCGGATTCAACCCCGAGGCAGCATCCATTCTAGAAAGAAAGTATCCATTTACAAAAGGGAAGTGGAACATCA
EF027139- IND-00-MH4        .........................T.........................................C......................
EU564335-CHIK31             .............A...........T.........................................C......................
EF210157-DRDE-06            .............A...........T.........................................C......................
EU244823-ITA07-RA1          .............A...........T...................................C.....C......................
EU037962 Wuerzburg 1        .............A...........T.........................................C......................
EF012359-D570/06            .............A...........T.........................................C......................
DQ443544-LR2006_OPY1        .............A...........T.........................................C......................
EU564334-TM25               .............A...........T.........................................C......................
EF027137-IND-06-RJ1         .............A...........T.........................................C......................
EF027136- IND-06-MH2        .............A...........T.........................................C......................
EF027134- IND-06-AP3        .............A...........T.........................................C......................
EF027135- IND-06-KA15       .............A...........T.........................................C......................
EF027138- IND-06-TN1        .............A...........T.........................................C......................
GQ428210 RGCB3-06           .............A...........T.........................................C......................
GQ428211 RGCB5-06           .............A...........T.........................................C......................
GQ428212 RGCB80-07          .............A...........T...................................C.....C......................
GQ428213 RGCB120-07         .............A...........T...................................C.....C......................
GQ428214 RGCB355-08         .............A...........T...................................C.....C......................
GQ428215 RGCB356-08         .............A...........T.........................................C......................
RGCB710-09                  .............A...........T.........................................C......................
RGCB711-09                  .............A...........T.........................................C......................
RGCB712-09                  .............A...........T....................C....................C......................
RGCB713-09                  .............A...........T.........................................C......................
RGCB715-09                  .............A...........T.........................................C......................
RGCB718-09                  .............A...........T.........................................C......................
RGCB721-09                  .............A...........T.........................................C......................
RGCB729-09                  .............A...........T.........................................C......................
RGCB730-09                  .............A...........T.........................................C......................
RGCB732-09                  .............A...........T.........................................C......................
RGCB734-09                  .............A...........T.........................................C......................
RGCB735-09                  .............A...........T.........................................C......................
RGCB736-09                  .............A......C....T.........................................C......................
RGCB739-09                  .............A...........T.........................................C......................
RGCB751-09                  .............A...........T.........................................C......................
RGCB754-09                  .............A...........T.........................................C......................
RGCB755-09                  .............A...........T.........................................C......................
RGCB756-09                  .............A...........T.........................................C......................
RGCB757-09                  .............A...........T.........................................C......................
RGCB-msq1-Chlym09           .............A...........T.........................................C......................
RGCB-msq2-Bpr09             .............A...........T.........................................C......................
RGCB-msq3-Olvna09           .............A...........T.........................................C......................
```

```
                          3430      3440      3450      3460      3470      3480      3490      3500      3510
                       ....|....|....|....|....|....|....|....|....|....|....|....|....|....|....|....|....|....|
AF369024- S27          ACAAGCAGATCTGCGTGACTACCAGGAGGATAGAAGACTTCAACCCTACCACCAACATTATACCGGCCAACAGGAGACTACCACACTCAT
EF027139- IND-00-MH4   ........................................T.................................................
EU564335-CHIK31        ........................................T.................C...............................
EF210157-DRDE-06       ........................................T.................C...............................
EU244823-ITA07-RA1     ........................................T..............T..C...............................
EU037962 Wuerzburg 1   ........................................T.................C...............................
EF012359-D570/06       ........................................T.................C...............................
DQ443544-LR2006_OPY1   ........................................T.................C...............................
EU564334-TM25          ........................................T.................C...............................
EF027137-IND-06-RJ1    ........................................T.................C...............................
EF027136- IND-06-MH2   ........................................T.................C...............................
EF027134- IND-06-AP3   ........................................T.................C...............................
EF027135- IND-06-KA15  ........................................T.................C...............................
EF027138- IND-06-TN1   ........................................T.................C...............................
GQ428210 RGCB3-06      ........................................T.................C...............................
GQ428211 RGCB5-06      ........................................T.................C...............................
GQ428212 RGCB80-07     ........................................T.................C...............................
GQ428213 RGCB120-07    ........................................T.................C...............................
GQ428214 RGCB355-08    ........................................T.................C...............................
GQ428215 RGCB356-08    ........................................T.................C...............................
RGCB710-09             ........................................T.................C...............................
RGCB711-09             ........................................T.................C...............................
RGCB712-09             ........................................T.................C...............................
RGCB713-09             ........................................T.................C...............................
RGCB715-09             ........................................T.................C...............................
RGCB718-09             ................A.......................T.................C...............................
RGCB721-09             ........................................T.................C...............................
RGCB729-09             ........................................T.................C...............................
RGCB730-09             ........................................T.................C...............................
RGCB732-09             ........................................T.................C...............................
RGCB734-09             ........................................T.................C...............................
RGCB735-09             ........................................T.................C...............................
RGCB736-09             ........................................T.................C...............................
RGCB739-09             ........................................T.................C...............................
RGCB751-09             ........................................T.................C...............................
RGCB754-09             ........................................T.................C...............................
RGCB755-09             ........................................T.................C...............................
RGCB756-09             ........................................T.................C...............................
RGCB757-09             ........................................T.................C...............................
RGCB-msq1-Chlym09      ........................................T.................C...............................
RGCB-msq2-Bpr09        ........................................T.................C...............................
RGCB-msq3-Olvna09      ........................................T.................C...............................
```

```
                                3520      3530      3540      3550      3560      3570      3580      3590      3600
                            ....|....|....|....|....|....|....|....|....|....|....|....|....|....|....|....|....|....|
AF369024- S27               TAGTGGCCGAACACCGCCCAGTAAAAGGGGAAAGAATGGAATGGCTGGTTAACAAGATAAACGGCCACCACGTGCTCCTGGTCAGTGGCT
EF027139- IND-00-MH4        ....................................................T.....................................
EU564335-CHIK31             ..........................................................................................
EF210157-DRDE-06            ..........................................................................................
EU244823-ITA07-RA1          ..........................................................................................
EU037962 Wuerzburg 1        ............................................N.............................................
EF012359-D570/06            ..........................................................................................
DQ443544-LR2006_OPY1        ..........................................................................................
EU564334-TM25               ..........................................................................................
EF027137-IND-06-RJ1         ..........................................................................................
EF027136- IND-06-MH2        ..........................................................................................
EF027134- IND-06-AP3        ..........................................................................................
EF027135- IND-06-KA15       ..........................................................................................
EF027138- IND-06-TN1        ..........................................................................................
GQ428210 RGCB3-06           ..........................................................................................
GQ428211 RGCB5-06           ..........................................................................................
GQ428212 RGCB80-07          ..........................................................................................
GQ428213 RGCB120-07         ..........................................................................................
GQ428214 RGCB355-08         ..........................................................................................
GQ428215 RGCB356-08         ..........................................................................................
RGCB710-09                  ..........................................................................................
RGCB711-09                  ..........................................................................................
RGCB712-09                  ..........................................................................................
RGCB713-09                  ..........................................................................................
RGCB715-09                  ..........................................................................................
RGCB718-09                  ..........................................................................................
RGCB721-09                  ..........................................................................................
RGCB729-09                  ..........................................................................................
RGCB730-09                  ..........................................................................................
RGCB732-09                  ..........................................................................................
RGCB734-09                  ..........................................................................................
RGCB735-09                  ..........................................................................................
RGCB736-09                  ..........................................................................................
RGCB739-09                  ..........................................................................................
RGCB751-09                  ..........................................................................................
RGCB754-09                  ..........................................................................................
RGCB755-09                  ..........................................................................................
RGCB756-09                  ..........................................................................................
RGCB757-09                  ..........................................................................................
RGCB-msq1-Chlym09           ..........................................................................................
RGCB-msq2-Bpr09             ..........................................................................................
RGCB-msq3-Olvna09           ..........................................................................................
```

```
                              3610      3620
                          ....|....|....|...
AF369024- S27             GTAGCCTTGCACTGCCTA
EF027139- IND-00-MH4      A..A..............
EU564335-CHIK31           A..A..............
EF210157-DRDE-06          A..A..............
EU244823-ITA07-RA1        A..A..............
EU037962 Wuerzburg 1      A..A..............
EF012359-D570/06          A..A..............
DQ443544-LR2006_OPY1      A..A..............
EU564334-TM25             A..A..............
EF027137-IND-06-RJ1       A..A..............
EF027136- IND-06-MH2      A..A..............
EF027134- IND-06-AP3      A..A..............
EF027135- IND-06-KA15     A..A..............
EF027138- IND-06-TN1      A..A..............
GQ428210 RGCB3-06         A..A..............
GQ428211 RGCB5-06         A..A..............
GQ428212 RGCB80-07        A..A..............
GQ428213 RGCB120-07       A..A..............
GQ428214 RGCB355-08       A..A..............
GQ428215 RGCB356-08       A..A..............
RGCB710-09                A..A..............
RGCB711-09                A..A..............
RGCB712-09                A..A..............
RGCB713-09                A..A..............
RGCB715-09                A..A..............
RGCB718-09                A..A..............
RGCB721-09                A..A..............
RGCB729-09                A..A..............
RGCB730-09                A..A..............
RGCB732-09                A..A..............
RGCB734-09                A..A..............
RGCB735-09                A..A..............
RGCB736-09                A..A..............
RGCB739-09                A..A..............
RGCB751-09                A..A..............
RGCB754-09                A..A..............
RGCB755-09                A..A..............
RGCB756-09                A..A..............
RGCB757-09                A..A..............
RGCB-msq1-Chlym09         A..A..............
RGCB-msq2-Bpr09           A..A..............
RGCB-msq3-Olvna09         A..A..............
```

1b. <u>E2 protein coding region</u>

```
                                     8900      8910      8920      8930      8940      8950      8960      8970      8980
                              ..|....|....|....|....|....|....|....|....|....|....|....|....|....|....|....|....|....|..
AF369024- S27                 AGTAGGAAGATTAGTCACTCATGTACGCACCCATTTCACCACGACCCTCCTGTGATAGGCCGGGAAAAATTCCATTCCCGACCGCAGCAC
EF027139- IND-00-MH4          ...........C..............................................................................
EU564335-CHIK31               .................T.........................................T..............................
EF210157-DRDE-06              .................T.........................................T..............................
EU244823-ITA07-RA1            .................T.........................................T..............................
EU037962 Wuerzburg 1          ...........................................................T..............................
EF012359-D570/06              ...........................................................T..............................
DQ443544-LR2006_OPY1          ...........................................................T..............................
EU564334-TM25                 ...........................................................T..............................
EF027137-IND-06-RJ1           .................T.........................................T..............................
EF027136- IND-06-MH2          .................T.........................................T..............................
EF027134- IND-06-AP3          .................T.........................................T..............................
EF027135- IND-06-KA15         .................T.........................................T..............................
EF027138- IND-06-TN1          .................T.........................................T..............................
GQ428210 RGCB3-06             .................T.........................................T..............................
GQ428211 RGCB5-06             .................T.........................................T..............................
GQ428212 RGCB80-07            .................T.........................................T..............................
GQ428213 RGCB120-07           ..C..............T.........................................T..............................
GQ428214 RGCB355-08           .................T.........................................T..............................
GQ428215 RGCB356-08           .................T.........................................T..............................
RGCB710-09                    .................T.........................................T..............................
RGCB711-09                    .................T........................C................T..............................
RGCB712-09                    .................T.........................................T..............................
RGCB713-09                    .................T.........................................T..............................
RGCB715-09                    .................T.........................................T..............................
RGCB718-09                    .................T.........................................T..............................
RGCB721-09                    .................T.........................................T..............................
RGCB729-09                    .................T.........................................T..............................
RGCB730-09                    .................T.........................................T..............................
RGCB732-09                    .................T......................C..................T..............................
RGCB734-09                    .................T.........................................T..............................
RGCB735-09                    .................T.........................................T..............................
RGCB736-09                    .................T.........................................T..............................
RGCB739-09                    .................T.........................................T..............................
RGCB751-09                    .................T.........................................T..............................
RGCB754-09                    .................T.........................................T..............................
RGCB755-09                    .................T.........................................T..............................
RGCB756-09                    .................T.....................T....T..............T..............................
RGCB757-09                    .................T.........................................T..............................
RGCB-msq1-Chlym09             .................T.........................................T..............................
RGCB-msq2-Bpr09               .................T.........................................T..............................
RGCB-msq3-Olvna09             .................T.........................................T..............................
```

```
                                     8990      9000      9010      9020      9030      9040      9050      9060      9070
                              ..|....|....|....|....|....|....|....|....|....|....|....|....|....|....|....|....|....|..
AF369024- S27                 GGTAAAGAGCTACCTTGCAGCACGTACGTGCAGAGCAACGCCGCAACTGCCGAGGAGATAGAGGTACACATGCCCCCAGACACCCCTGAT
EF027139- IND-00-MH4          .....................................C.....T....A.........................................
EU564335-CHIK31               .....................................C..........A.........................................
EF210157-DRDE-06              .....................................C..........A.........................................
EU244823-ITA07-RA1            .....................................C..........A.........................................
EU037962 Wuerzburg 1          .....................................C..........A.........................................
EF012359-D570/06              .....................................C..........A.........................................
DQ443544-LR2006_OPY1          .....................................C..........A.........................................
EU564334-TM25                 .....................................C..........A.........................................
EF027137-IND-06-RJ1           .....................................C..........A.........................................
EF027136- IND-06-MH2          .....................................C..........A.........................................
EF027134- IND-06-AP3          .....................................C..........A.........................................
EF027135- IND-06-KA15         .....................................C..........A.........................................
EF027138- IND-06-TN1          .....................................C..........A.........................................
GQ428210 RGCB3-06             .....................................C..........A.........................................
GQ428211 RGCB5-06             .....................................C..........A.........................................
GQ428212 RGCB80-07            .....................................C..........A.........................................
GQ428213 RGCB120-07           .....................................C..........A.........................................
GQ428214 RGCB355-08           .....................................C..........A.........................................
GQ428215 RGCB356-08           .....................................C..........A.........................................
RGCB710-09                    .....................................C..........A.........................................
RGCB711-09                    .....................................C..........A.........................................
RGCB712-09                    .....................................C..........A.........................................
RGCB713-09                    .....................................C..........A.........................................
RGCB715-09                    .....................................C..........A.........................................
RGCB718-09                    .....................................C..........A.........................................
RGCB721-09                    .....................................C..........A.........................................
RGCB729-09                    .....................................C..........A.........................................
RGCB730-09                    .....................................C..........A.........................................
RGCB732-09                    .....................................C..........A.........................................
RGCB734-09                    .....................................C..........A.........................................
RGCB735-09                    .....................................C..........A.........................................
RGCB736-09                    .....................................C..........A.........................................
RGCB739-09                    .....................................C..........A.........................................
RGCB751-09                    .....................................C..........A.........................................
RGCB754-09                    .....................................C..........A.........................................
RGCB755-09                    .....................................C..........A.........................................
RGCB756-09                    .....................................C..........A.........................................
RGCB757-09                    .....................................C..........A.........................................
RGCB-msq1-Chlym09             .....................................C..........A.........................................
RGCB-msq2-Bpr09               .....................................C..........A.........................................
RGCB-msq3-Olvna09             .....................................C..........A.........................................
```

```
                                   9080      9090      9100      9110      9120      9130      9140      9150      9160
                             ..|....|....|....|....|....|....|....|....|....|....|....|....|....|....|....|....|....|..
AF369024- S27                CGCACATTGCTGTCACAACAGTCCGGCAACGTAAAGATCACAGTCAATAGTCAGACGGTGCGGTATAAGTGTAATTGCGGTGGCTCAAAT
EF027139- IND-00-MH4         .........A..................................T...G.C..............C........................
EU564335-CHIK31              ........AA......................................G.C..............C........................
EF210157-DRDE-06             ........AA......................................G.C..............C........................
EU244823-ITA07-RA1           ........AA......................................G.C..............C........................
EU037962 Wuerzburg 1         ........AA......................................G.C..............C........................
EF012359-D570/06             ........AA......................................G.C..............C........................
DQ443544-LR2006_OPY1         ........AA......................................G.C..............C........................
EU564334-TM25                ........AA......................................G.C..............C........................
EF027137-IND-06-RJ1          ........AA......................................G.C..............C........................
EF027136- IND-06-MH2         ........AA......................................G.C..............C........................
EF027134- IND-06-AP3         ........AA......................................G.C..............C........................
EF027135- IND-06-KA15        ........AA......................................G.C..............C........................
EF027138- IND-06-TN1         ........AA......................................G.C..............C........................
GQ428210 RGCB3-06            ........AA......................................G.C..............C........................
GQ428211 RGCB5-06            ........AA......................................G.C..............C........................
GQ428212 RGCB80-07           ........AA......................................G.C..............C........................
GQ428213 RGCB120-07          ........AA......................................G.C..............C........................
GQ428214 RGCB355-08          ........AA......................................G.C..............C........................
GQ428215 RGCB356-08          ........AA...............................G......G.C..............C........................
RGCB710-09                   ........AA...............................G......G.C..............C........................
RGCB711-09                   ........AA...............................G......G.C..............C........................
RGCB712-09                   ........AA...............................G......G.C..............C........................
RGCB713-09                   ........AA...............................G......G.C..............C........................
RGCB715-09                   ........AA...............................G......G.C..............C........................
RGCB718-09                   ........AA...............................G......G.C..............C........................
RGCB721-09                   ........AA...............................G......G.C..............C........................
RGCB729-09                   ........AA...............................G......G.C..............C........................
RGCB730-09                   ......C.AA...............................G......G.C..............C........................
RGCB732-09                   ........AA...............................G......G.C..............C........................
RGCB734-09                   ........AA...............................G......G.C..............C........................
RGCB735-09                   ........AA...............................G......G.C..............C........................
RGCB736-09                   ........AA...............................G......G.C..............C........................
RGCB739-09                   ........AA...............................G......G.C.......A......C........................
RGCB751-09                   ........AA...............................G......G.C..............C........................
RGCB754-09                   ........AA...............................G......G.C..............C........................
RGCB755-09                   ........AA...............................G......G.C..............C........................
RGCB756-09                   ........AA...............................G......G.C..A...........C........................
RGCB757-09                   ........AA...............................G......G.C..............C........................
RGCB-msq1-Chlym09            ........AA...............................G......G.C..............C........................
RGCB-msq2-Bpr09              ........AA...............................G......G.C..............C........................
RGCB-msq3-Olvna09            ........AA...............................G......G.C..............C........................
```

```
                           9170      9180      9190      9200      9210      9220      9230      9240      9250
                       ..|....|....|....|....|....|....|....|....|....|....|....|....|....|....|....|....|....|..
AF369024- S27          GAAGGACTAATAACTACAGATAAAGTGATTAATAACTGCAAGGTTGATCAATGTCATGCCGCGGTCACCAATCACAAAAAGTGGCAGTAT
EF027139- IND-00-MH4   ....................C.....................................................................
EU564335-CHIK31        ..........C.........C.....................................................................
EF210157-DRDE-06       ..........C.........C.....................................................................
EU244823-ITA07-RA1     ..........C.........C.....................................................................
EU037962 Wuerzburg 1   ..........C.........C.....................................................................
EF012359-D570/06       ..........C.........C.....................................................................
DQ443544-LR2006_OPY1   ..........C.........C.....................................................................
EU564334-TM25          ..........C.........C.....................................................................
EF027137-IND-06-RJ1    ..........C.........C.....................................................................
EF027136- IND-06-MH2   ..........C.........C.....................................................................
EF027134- IND-06-AP3   ..........C.........C.....................................................................
EF027135- IND-06-KA15  ..........C.........C.....................................................................
EF027138- IND-06-TN1   ..........C.........C.....................................................................
GQ428210 RGCB3-06      ..........C.........C.....................................................................
GQ428211 RGCB5-06      ..........C.........C.....................................................................
GQ428212 RGCB80-07     ..........C.........C.....................................................................
GQ428213 RGCB120-07    ..........C.........C.....................................................................
GQ428214 RGCB355-08    ..........C.........C.....................................................................
GQ428215 RGCB356-08    ..........C.........C..........................................A..........................
RGCB710-09             .......A..C.........C.....................................................................
RGCB711-09             .......A..C.........C.....................................................................
RGCB712-09             .......A..C.........C.....................................................................
RGCB713-09             .......A..C.........C.....................................................................
RGCB715-09             .......A..C.........C.....................................................................
RGCB718-09             .......A..C.........C.....................................................................
RGCB721-09             .......A..C.........C.....................................................................
RGCB729-09             .......A..C.........C.....................................................................
RGCB730-09             .......A..C.........C.....................................................................
RGCB732-09             .......A..C.........C.....................................................................
RGCB734-09             .......A..C.........C.....................................................................
RGCB735-09             .......A..C.........C.....................................................................
RGCB736-09             .......A..C.........C.....................................................................
RGCB739-09             .......A..C.........C...............................................................A.....
RGCB751-09             .......A..C.........C.....................................................................
RGCB754-09             .......A..C.........C.....................................................................
RGCB755-09             .......A..C.........C.....................................................................
RGCB756-09             .......A..C.........C.....................................................................
RGCB757-09             .......A..C.........C...................................................................C.
RGCB-msq1-Chlym09      .......A..C.........C.....................................................................
RGCB-msq2-Bpr09        .......A..C.........C.....................................................................
RGCB-msq3-Olvna09      .......A..C.........C.....................................................................
```

```
                              9260      9270      9280      9290      9300      9310      9320
                         ..|....|....|....|....|....|....|....|....|....|....|....|....|....|
AF369024- S27            AACTCCCCTCTGGTCCCGCGTAACGCTGAACTCGGGGACCGAAAAGGAAAAATTCACATCCCGTTTCC
EF027139- IND-00-MH4     .......................T........T...................................
EU564335-CHIK31          .......................T........T...................................
EF210157-DRDE-06         .......................T........T...................................
EU244823-ITA07-RA1       .......................T........T...................................
EU037962 Wuerzburg 1     .......................T........T...................................
EF012359-D570/06         .......................T........T...................................
DQ443544-LR2006_OPY1     .......................T........T...................................
EU564334-TM25            .......................T........T...................................
EF027137-IND-06-RJ1      .......................T........T...................................
EF027136- IND-06-MH2     .......................T........T...................................
EF027134- IND-06-AP3     .......................T........T...................................
EF027135- IND-06-KA15    .......................T........T...................................
EF027138- IND-06-TN1     .......................T........T...................................
GQ428210 RGCB3-06        .......................T........T...................................
GQ428211 RGCB5-06        .......................T........T...................................
GQ428212 RGCB80-07       .......................T........T.........C.........................
GQ428213 RGCB120-07      .......................T........T.........C.........................
GQ428214 RGCB355-08      .......................T........T.........C.........................
GQ428215 RGCB356-08      .......................T........T...................................
RGCB710-09               .......................T........T...................................
RGCB711-09               .......................T........T...................................
RGCB712-09               .......................T........T...................................
RGCB713-09               .......................T........T...................................
RGCB715-09               .......................T........T...................................
RGCB718-09               .......................T........T...................................
RGCB721-09               .......................T........T...................................
RGCB729-09               .......................T........T...................................
RGCB730-09               .......................T........T...................................
RGCB732-09               .......................T........T...................................
RGCB734-09               .......................T........T...................................
RGCB735-09               .......................T........T...................................
RGCB736-09               .......................T........T...................................
RGCB739-09               .......................T........T...................................
RGCB751-09               .......................T........T...................................
RGCB754-09               .......................T........T...................................
RGCB755-09               .......................T........T...................................
RGCB756-09               .......................T........T...................................
RGCB757-09               .......................T........T...................................
RGCB-msq1-Chlym09        .......................T........T...................................
RGCB-msq2-Bpr09          .......................T........T...................................
RGCB-msq3-Olvna09        .......................T........T...................................
```

1c. E1 protein coding region

```
                              10430     10440     10450     10460     10470     10480     10490     10500     10510
                           ...|....|....|....|....|....|....|....|....|....|....|....|....|....|....|....|....|....|.
AF369024- S27              CTGCCTATGCAAACGGCGACCATGCCGTCACAGTTAAGGACGCCAAATTCATTGTGGGGCCAATGTCTTCAGCCTGGACACCTTTTGACA
EF027139- IND-00-MH4       ..................................C..................................................C....
EU564335-CHIK31            .....................................................................................C....
EF210157-DRDE-06           .....................................................................................C....
EU244823-ITA07-RA1         .....................................................................................C....
EU037962 Wuerzburg 1       .....................................................................................C....
EF012359-D570/06           .....................................................................................C....
DQ443544-LR2006_OPY1       .....................................................................................C....
EU564334-TM25              .....................................................................................C....
EF027137-IND-06-RJ1        .....................................................................................C....
EF027136- IND-06-MH2       .....................................................................................C....
EF027134- IND-06-AP3       .....................................................................................C....
EF027135- IND-06-KA15      .....................................................................................C....
EF027138- IND-06-TN1       .....................................................................................C....
GQ428210 RGCB3-06          .....................................................................................C....
GQ428211 RGCB5-06          .....................................................................................C....
GQ428212 RGCB80-07         .....................................................................................C....
GQ428213 RGCB120-07        .....................................................................................C.G..
GQ428214 RGCB355-08        .....................................................................................C....
GQ428215 RGCB356-08        .....................................................................................C....
RGCB710-09                 .....................................................................................C....
RGCB711-09                 .....................................................................................C....
RGCB712-09                 .....................................................................................C....
RGCB713-09                 .....................................................................................C....
RGCB715-09                 .....................................................................................C....
RGCB718-09                 .....................................................................................C....
RGCB721-09                 .....................................................................................C....
RGCB729-09                 .....................................................................................C....
RGCB730-09                 .....................................................................................C....
RGCB732-09                 .....................................................................................C....
RGCB734-09                 .....................................................................................C....
RGCB735-09                 .....................................................................................C....
RGCB736-09                 .....................................................................................C....
RGCB739-09                 .....................................................................................C....
RGCB751-09                 .....................................................................................C....
RGCB754-09                 .....................................................................................C....
RGCB755-09                 .....................................................................................C....
RGCB756-09                 .....................................................................................C....
RGCB757-09                 .....................................................................................C....
RGCB-msq1-Chlym09          .....................................................................................C....
RGCB-msq2-Bpr09            .............................-................-......................................C....
RGCB-msq3-Olvna09          .....................................................................................C....
```

```
                          10520     10530     10540     10550     10560     10570     10580     10590     10600
                        ...|....|....|....|....|....|....|....|....|....|....|....|....|....|....|....|....|....|.
AF369024- S27           ACAAAATCGTGGTGTACAAAGGTGACGTTTACAACATGGACTACCCGCCCTTTGGCGCAGGAAGACCAGGACAATTTGGCGATATCCAAA
EF027139- IND-00-MH4    .......T....................C..T.................T........................................
EU564335-CHIK31         .......T....................C..T..........................................................
EF210157-DRDE-06        .......T....................C..T..........................................................
EU244823-ITA07-RA1      .......T....................C..T..........................................................
EU037962 Wuerzburg 1    .......T....................C..T..........................................................
EF012359-D570/06        .......T....................C..T..........................................................
DQ443544-LR2006_OPY1    .......T....................C..T..........................................................
EU564334-TM25           .......T....................C..T..........................................................
EF027137-IND-06-RJ1     .......T....................C..T..........................................................
EF027136- IND-06-MH2    .......T....................C..T..........................................................
EF027134- IND-06-AP3    .......T....................C..T..........................................................
EF027135- IND-06-KA15   .......T....................C..T..........................................................
EF027138- IND-06-TN1    .......T....................C..T..........................................................
GQ428210 RGCB3-06       .......T....................CC.T..........................................................
GQ428211 RGCB5-06       .......T.......................T...G......................................................
GQ428212 RGCB80-07      .......T....................C..T..........................................................
GQ428213 RGCB120-07     .......T....................C..T..........................................................
GQ428214 RGCB355-08     .......T....................C..T..........................................................
GQ428215 RGCB356-08     .......T....................C..T..........................................................
RGCB710-09              .......T....................C..T..........................................................
RGCB711-09              .......T....................C..T..........................................................
RGCB712-09              .......T....................C..T..........................................................
RGCB713-09              .......T....................C..T..........................................................
RGCB715-09              .......T....................C..T..........................................................
RGCB718-09              .......T....................C..T..........................................................
RGCB721-09              .......T....................C..T..........................................................
RGCB729-09              .......T....................C..T..........................................................
RGCB730-09              .......T....................C..T..........................................................
RGCB732-09              .......T....................C..T..........................................................
RGCB734-09              .......T....................C..T..........................................................
RGCB735-09              .......T....................C..T..........................................................
RGCB736-09              .......T....................C..T..........................................................
RGCB739-09              .......T....................C..T..........................................................
RGCB751-09              .......T....................C..T..........................................................
RGCB754-09              .......T....................C..T..........................................................
RGCB755-09              .......T....................C..T..........................................................
RGCB756-09              .......T....................C..T..........................................................
RGCB757-09              .......T....................C..T..........................................................
RGCB-msq1-Chlym09       .......T....................C..T..........................................................
RGCB-msq2-Bpr09         .......T....................C..T..........................................................
RGCB-msq3-Olvna09       .......T....................C..T..........................................................
```

```
                          10610     10620     10630     10640     10650     10660     10670     10680     10690
                        ...|....|....|....|....|....|....|....|....|....|....|....|....|....|....|....|....|....|.
AF369024- S27           GTCGCACGCCTGAGAGCAAAGACGTCTATGCTAACACACAACTGGTACTGCAGAGACCGGCTGCGGGTACGGTACACGTGCCATACTCTC
EF027139- IND-00-MH4    .......A........T.................T.........................................T.............
EU564335-CHIK31         .......A........T.................T.......................................................
EF210157-DRDE-06        .......A........T.................T.......................................................
EU244823-ITA07-RA1      .......A........T.................T............................T..........................
EU037962 Wuerzburg 1    .......A........T.................T............................T..........................
EF012359-D570/06        .......A........T.................T............................T..........................
DQ443544-LR2006_OPY1    .......A........T.................T............................T..........................
EU564334-TM25           .......A........T.................T............................T..........................
EF027137-IND-06-RJ1     .......A........T.................T.......................................................
EF027136- IND-06-MH2    .......A........T.................T.......................................................
EF027134- IND-06-AP3    .......A........T.................T.......................................................
EF027135- IND-06-KA15   .......A........T..C..............T.......................................................
EF027138- IND-06-TN1    .......A........T......A..........T.......................................................
GQ428210 RGCB3-06       .......A........T.................T.......................................................
GQ428211 RGCB5-06       .......A........T.................T.......................................................
GQ428212 RGCB80-07      .......A........T.................T............................T..........................
GQ428213 RGCB120-07     .......A........T.................T............................T..........................
GQ428214 RGCB355-08     .......A........T.................T............................T..........................
GQ428215 RGCB356-08     .......A........T.................T............................T..........................
RGCB710-09              .......A........T.................T............................T..........................
RGCB711-09              .......A........T.................T............................T..........................
RGCB712-09              .......A........T.................T............................T..........................
RGCB713-09              .......A........T.................T............................T..........................
RGCB715-09              .......A........T.................T............................T..........................
RGCB718-09              .......A........T.................T............................T..........................
RGCB721-09              .......A........T.................T............................T..........................
RGCB729-09              .......A........T.................T............................T..........................
RGCB730-09              .......A........T.................T............................T..........................
RGCB732-09              .......A........T.................T............................T..........................
RGCB734-09              .......A........T.................T............................T..........................
RGCB735-09              .......A........T.................T............................T..........................
RGCB736-09              .......A........T.................T............................T..........................
RGCB739-09              .......A........T.................T............................T..........................
RGCB751-09              .......A........T.................T............................T..........................
RGCB754-09              .......A........T.................T............................T..........................
RGCB755-09              .......A........T.................T............................T..........................
RGCB756-09              .......A........T.................T............................T..........................
RGCB757-09              .......A........T.................T............................T..........................
RGCB-msq1-Chlym09       .......A........T.................T............................T..........................
RGCB-msq2-Bpr09         .......A........T.................T............................T..........................
RGCB-msq3-Olvna09       .......A........T.................T............................T..........................
```

```
                       10700     10710     10720     10730     10740     10750     10760     10780     10780
                      ...|....|....|....|....|....|....|....|....|....|....|....|....|....|....|....|....|....|.
AF369024- S27         AGGCACCATCTGGCTTTAAGTATTGGTTAAAAGAACGAGGGGCGTCGCTACAGCACACAGCACCATTTGGCTGCCAAATAGCAACAAACC
EF027139- IND-00-MH4  ..........................C.......................G........................................
EU564335-CHIK31       ..........................C..........C........A..G........................................
EF210157-DRDE-06      ..........................C..........C........A..G........................................
EU244823-ITA07-RA1    ..........................C..........C........A..G........................................
EU037962 Wuerzburg 1  ..........................C..........C...........G........................................
EF012359-D570/06      ..........................C..........C...........G........................................
DQ443544-LR2006_OPY1  ..........................C..........C...........G........................................
EU564334-TM25         ..........................C..........C...........G........................................
EF027137-IND-06-RJ1   ..........................C..........C........A..G........................................
EF027136- IND-06-MH2  ..........................C..........C...........G........................................
EF027134- IND-06-AP3  ..........................C..........C........A..G........................................
EF027135- IND-06-KA15 ..........................C..........C...........G........................................
EF027138- IND-06-TN1  ..........................C..........C........A..G........................................
GQ428210 RGCB3-06     ..........................C..........C........A..G........................................
GQ428211 RGCB5-06     ..........................C..........C........A..G........................................
GQ428212 RGCB80-07    ..........................C.......-..C........A..G........................................
GQ428213 RGCB120-07   ..........................C..........C........A..G........................................
GQ428214 RGCB355-08   ..........................C..........C.A......A..G........................................
GQ428215 RGCB356-08   ..........................C..........C........A..G........................................
RGCB710-09            ..........................C..........C........A..G........................................
RGCB711-09            ..........................C..........C........A..G........................................
RGCB712-09            ..........................C..........C........A..G........................................
RGCB713-09            ..........................C..........C........A..G........................................
RGCB715-09            ..........................C..........C........A..G........................................
RGCB718-09            ..........................C..........C........A..G..............G.........................
RGCB721-09            ..........................C..........C........A..G........................................
RGCB729-09            ..........................C..........C........A..G........................................
RGCB730-09            ..........................C..........C........A..G........................................
RGCB732-09            ..........................C..........C........A..G........................................
RGCB734-09            ..........................C..........C........A..G........................................
RGCB735-09            ..........................C..........C........A..G........................................
RGCB736-09            ..........................C..........C........A..G........................................
RGCB739-09            ..........................C..........C........A..G........................................
RGCB751-09            ..........................C..........C........A..G........................................
RGCB754-09            ..........................C..........C........A..G........................................
RGCB755-09            ..........................C..........C........A..G........................................
RGCB756-09            ..........................C..........C........A..G........................................
RGCB757-09            ..........................C..........C........A..G........................................
RGCB-msq1-Chlym09     ..........................C..........C........A..G........................................
RGCB-msq2-Bpr09       ..........................C..........C........A..G........................................
RGCB-msq3-Olvna09     ..........................C..........C........A..G........................................
```

```
                             10790     10800     10810     10820     10830     10840     10850     10860     10870
                            ...|....|....|....|....|....|....|....|....|....|....|....|....|....|....|....|....|....|.
AF369024- S27               CGGTAAGAGCGATGAACTGCGCCGTAGGGAACATGCCCATCTCCATCGACATACCGGATGCGGCCTTCACTAGGGTCGTCGACGCGCCCT
EF027139- IND-00-MH4        ...........G.......................................................T......................
EU564335-CHIK31             ...........G..............................................A...............................
EF210157-DRDE-06            ...........G..............................................A...............................
EU244823-ITA07-RA1          ...........G..............................................A...............................
EU037962 Wuerzburg 1        ...........G..............................................A...............................
EF012359-D570/06            ...........G..............................................A...............................
DQ443544-LR2006_OPY1        ...........G..............................................A...............................
EU564334-TM25               ...........G..............................................A...............................
EF027137-IND-06-RJ1         ...........G..............................................A...............................
EF027136- IND-06-MH2        ...........G..............................................A...............................
EF027134- IND-06-AP3        ...........G..............................................A...............................
EF027135- IND-06-KA15       ...........G..............................................A...............................
EF027138- IND-06-TN1        ...........G..............................................A...............................
GQ428210 RGCB3-06           ...........G.............G................................A...............................
GQ428211 RGCB5-06           ...........G..............................................A...............................
GQ428212 RGCB80-07          ...........G..............................................A...............................
GQ428213 RGCB120-07         ...........G..............................................A...............................
GQ428214 RGCB355-08         ...........G..............................................A...............................
GQ428215 RGCB356-08         ...........G..............................................A...............................
RGCB710-09                  ...........G..............................................A...............................
RGCB711-09                  ...........G..............................................A..................A............
RGCB712-09                  ...........G..............................................A...............................
RGCB713-09                  ...........G..............................................A...............................
RGCB715-09                  ...........G..............................................A...............................
RGCB718-09                  ...........G..............................................A...............................
RGCB721-09                  ...........G..............................................A...............................
RGCB729-09                  ...........G..............................................A...............................
RGCB730-09                  ...........G..............................................A...............................
RGCB732-09                  ...........G..............................................A...............................
RGCB734-09                  ...........G..............................................A...............................
RGCB735-09                  ...........G..............................................A...............................
RGCB736-09                  ...........G..............................................A...............................
RGCB739-09                  ...........G..............................................A...............................
RGCB751-09                  ...........G..............................................A...............................
RGCB754-09                  ...........G..............................................A...............................
RGCB755-09                  ...........G..............................................A..................A............
RGCB756-09                  ...........G..............................................A...............................
RGCB757-09                  ...........G..............................................A...............................
RGCB-msq1-Chlym09           ...........G..............................................A..................A............
RGCB-msq2-Bpr09             ...........G..............................................A..................A............
RGCB-msq3-Olvna09           ...........G..............................................A..................A............
```

```
                              10880     10890     10900     10910     10920     10930
                            ...|....|....|....|....|....|....|....|....|....|....|.
AF369024- S27               CTTTAACGGACATGTCATGCGAGGTACCAGCCTGCACCCATTCCTCAGACTTTGG
EF027139- IND-00-MH4        ................G................................T..C..
EU564335-CHIK31             ................G......................................
EF210157-DRDE-06            ................G......................................
EU244823-ITA07-RA1          ................G......................................
EU037962 Wuerzburg 1        ................G......................................
EF012359-D570/06            ................G......................................
DQ443544-LR2006_OPY1        ................G......................................
EU564334-TM25               ................G......................................
EF027137-IND-06-RJ1         ................G......................................
EF027136- IND-06-MH2        ................G......................................
EF027134- IND-06-AP3        ................G......................................
EF027135- IND-06-KA15       ................G......................................
EF027138- IND-06-TN1        ................G......................................
GQ428210 RGCB3-06           ................G......................................
GQ428211 RGCB5-06           ................G......................................
GQ428212 RGCB80-07          ................G......................................
GQ428213 RGCB120-07         ................G......................................
GQ428214 RGCB355-08         ................G......................................
GQ428215 RGCB356-08         ................G..........T...........................
RGCB710-09                  ................G......................................
RGCB711-09                  ................G......................................
RGCB712-09                  .......A........G......................................
RGCB713-09                  ................G......................................
RGCB715-09                  ................G......................................
RGCB718-09                  ................G......................................
RGCB721-09                  ................G......................................
RGCB729-09                  ................G......................................
RGCB730-09                  ................G......................................
RGCB732-09                  ................G......................................
RGCB734-09                  ................G......................................
RGCB735-09                  ................G......................................
RGCB736-09                  ................G......................................
RGCB739-09                  ................G......................................
RGCB751-09                  ................G......................................
RGCB754-09                  ................G......................................
RGCB755-09                  ................G......................................
RGCB756-09                  ................G......................................
RGCB757-09                  ................G......................................
RGCB-msq1-Chlym09           ................G......................................
RGCB-msq2-Bpr09             ................G......................................
RGCB-msq3-Olvna09           ................G......................................
```
